# Time preference and personal value: a population-based cross-sectional study in Japan

**DOI:** 10.1186/s40359-020-00458-6

**Published:** 2020-08-17

**Authors:** Norito Kawakami, Kazuhiro Watanabe, Daisuke Nishi, Daisuke Takagi, Hideki Hashimoto, Saori C. Tanaka

**Affiliations:** 1 Department of Mental Health, Graduate School of Medicine, The University of Tokyo, Tokyo, Japan; 2 Department of Health and Social Behavior, Graduate School of Medicine, The University of Tokyo, Tokyo, Japan; 3 ATR Brain Information Communication Research Laboratory Group, Kyoto, Japan

**Keywords:** Time discount, Hyperbolic time discount, Priority value, Commitment to value, Community population

## Abstract

**Objective:**

This study aimed to investigate the association between time preference (i.e., time discounting and hyperbolic time discounting) and personal values (the areas of priority values and commitment to value) in a sample of adult community residents in Japan.

**Methods:**

Data from respondents (*N* = 2787) who completed the wave 1 and 3 surveys of a three-wave panel study of adult community residents in municipalities in Tokyo and suburban areas spanning 2010–2017 were analysed. Time discount rate and hyperbolic discount were measured using a three-item choice-based scale at the wave 1. Areas of priority value at present and at age 15 were measured by 11 questions for different value areas at the wave 3; the commitment to value at present and age 15 was measured by the Personal Value Questionnaire-II (PVQ-II) at the wave 3. Linear regression analyses were conducted of priority areas of values and commitment to value on time preference indicators, adjusting for sociodemographic variables and childhood socioeconomic status.

**Results:**

After excluding those with missing responses, data from 1880 and 1958 respondents were subject to analyses on time discounting and hyperbolic time discounting, respectively. Time discount rate was significantly and negatively associated with the value area of maintaining a stable life at present. Hyperbolic time discounting was significantly and negatively associated with the commitment to value at age 15.

**Conclusion:**

There may be an association between time preference and personal values. Time discounting and hyperbolic time discounting may be associated with different aspects of personal values, i.e., area of priority values and commitment to value, respectively.

## Background

Personal values are defined as broad, desirable, and trans-situational goals and underlie and guide attitudes and behaviour of individuals [1]. Research has distinguished two concepts of personal value, but both are associated with health and well-being: the content of values [2], that are areas of priority values or motivational types of values for an individual, and the commitment to value [3], that is the degree that an individual perceives a certain value important and behaves aiming at it as a goal. Previous studies reported that the areas of priority values are associated with various health outcomes and psychological well-being [4–7], as well as other important consequences, such as one’s identity, career choice, attitudes and behavior toward people [2]. The commitment to value was also associated with well-being [8, 9]. Thus personal value may have a public health significance in preventing and promoting health and well-being of people. However the determinants and health impacts of personal value are still largely understudied [1].

A number of variables may affect the acquisition and character of personal value. For example, gender and age are associated with personal value [8, 10]. Moreover, personality traits are thought to be key components in the development of personal value. A previous meta-analysis showed the association between Big Five personality traits and the areas of priority values: openness to experience is associated with self-direction, stimulation and universalism; agreeableness with benevolence and universalism; and extraversion with power, achievement orientation and stimulation seeking [11]. However, other personal psychological factors may also be associated with personal values.

Time preference is the value-related tendency of individuals to choose intertemporal options based on a value or expected reward [12]. Among several related concepts, time discounting is a tendency that people discount delayed rewards as they become distant in time and are perceived less valuable. In other words, people with this tendency give greater value to immediate rewards than delayed rewards. The degree of time discounting often refers impulsiveness or impatience. Time discounting is known to affect people’s economic behaviours [12], including debt holdings [13]. It was also associated with high-risk health-related behaviours, such as poor diet, obesity [12–15] and smoking [15–18]. While time discounting model assumes that the discounting rate is constant across time (ex. exponential discounting model), there are several anomalies. The most well-known anomaly is hyperbolic discounting [12, 19]. This is a time-inconsistent model of time discounting with a decreasing rate of time discounting: the discount rates implied in two recent dates are higher than that of two remote dates. Hyperbolic time discounting is thought to indicate the tendency of procrastination [12]. Hyperbolic discounting are also known to explain people’s economic behaviours, such as wealth accumulation and credit card borrowing [19], and also to be associated with high-risk health-related behaviours, such as smoking [20] and obesity [13, 15]. Interestingly, time discounting and hyperbolic time discounting were independently associated with these poor health-related behaviours [15]. In human neuroeconomic studies, time discounting has been associated with a balance or crosstalk between two neural systems: one consists of neural areas exhibited reward-related activity (ventral striatum, medial prefrontal cortex, posterior cingulate cortex) associated with choice of immediate reward or larger discount rate [21–26], and the other is an “executive attention network” (dorsolateral prefrontal cortex, dorsomedial prefrontal cortex, inferior parietal cortex) associated with choice of delayed reward [21, 25, 27, 28]. Previous studies have suggested that immature of executive attention network in terms of the structural connectivity [28], brain volume [27], and functional activation [25] or dominance of reward-related system [22, 25] might cause greater delay discounting observed in adolescent. It is said that hyperbolic time discount is also explained by the combined activity of these two systems [24].

Time preference could also be associated with personal value. It may be hypothesised that time discounting could be associated with placing a lower priority on values that involve a long-term goal due to high levels of impulsiveness or impatience [12]. Hyperbolic time discounting, meanwhile, may be associated with a less tendency to stick to a certain value area, since people with this tendency show time inconsistency of choices [12] and they perceive that there could be an alternative better value that they should commit to in the future. Thus, people with this tendency may show a lower commitment to value. Second, as time preference changes over time as prefrontal regulatory regions mature [22, 25], personal value may affect the age-related maturation of time preference (i.e., impatience). For instance, having a value priority for areas related to secure development and maturation of human may be promoting healthy development of the forebrain and thus associated with non-deviated time preference patterns. Positive emotions and wellbeing that stem from healthy value priorities could also affect time preference [29]. Third, personal value is thought to develop as part of the psychological process in which adolescents acquire the ability to control the conflict between learned values and actual behaviour by using self-regulation [30]. Time preference are also supposed to establish in adolescence after puberty and gradually develop from adolescence to adulthood [22]. These two bases of human behaviours may co-develop over time, interacting each other. Furthermore, if a specific pattern of the association between the components of personal values and time preference exits, if any, it would contribute to further understanding of mechanisms establishing personal values.

As the first step to know the relationship between personal values and time preference, the present study aims to investigate the cross-sectional associations of time preference (time discounting and hyperbolic time discounting) with personal values (the areas of priority values and commitment to value) at present assessed by a self-report questionnaire in a large community adult sample in Japan. Although the present study was cross-sectional, we also attempted to measure personal values at age 15 using a retrospective recall and associated the personal values at age 15 with time preference, because the association between time preference and personal values in adolescence may be clearer before both tendencies are later modified by experiences in adulthood. We hypothesized that time preference (time discounting) could be negatively associated with value priorities for long-term goals while hyperbolic time discounting could be negatively associated with the commitment to value. The pattern may be more prominent for personal values at age 15 than for that in adulthood (at the survey).

## Methods

### Sample

The Japanese Study on Stratification, Health, Income and Neighbourhood (J-SHINE) is a longitudinal panel study of young and middle-aged (aged 25–50 years old) community residents in the greater Tokyo metropolitan area of Japan [31]. The wave 1 survey was carried out in 2010. The survey participants were re-contacted in 2012 and 2017 (waves 2 and 3). A computer-based self-report questionnaire (wave 1 and 2) and a self-administered questionnaire (wave 3) were used to collect information covering socio-demographics, behavioural tendencies, personal values and developmental history. A total of 4385 respondents participated in the wave 1 survey (response rate: 31.3%). A total of 2787 individuals (63.6% of the wave 1 respondents) responded to the wave 3 survey. In this study, we used data from respondents who completed both wave 1 and wave 3 surveys, because the wave 1 survey collected information of time preference, and the wave 3 survey asked questions of personal values.

### Measures

#### Time preference

Time discounting and hyperbolic time discounting were measured with the wave 1 questionnaire, by using a three-item choice-based measure as described in an earlier study using the same dataset [18], adopting a scale developed by previous studies [13, 15], with applying a common methodology for large-scale studies [32, 33] (see Additional file 1: Appendix 1). Participants were asked to complete three questions in which they made a series of binary choices between receiving a smaller amount of money sooner or a larger amount of money. Q1 asked participants to choose between receiving 10,000 yen in 1 month (option A) or receiving more (an amount which increased from 9500 to more than 14,000 yen via nine steps) in 13 months (option B). Q2 asked respondents to choose between receiving 1 million yen in 1 month (option A) or receiving more (an amount which increased from 0.95 million to more than 1.4 million yen via nice points) in 13 months (option B). Q3 asked participants to choose between receiving 1 million yen in 13 months (option A) or receiving more (increasing from 0.95 million to more than 1.4 million yen via nice points) in 25 months (option B). If a respondent switched from option A to option B, he or she was given an interest rate (equivalent to the amount of money for option B divided by the amount of money for option A minus 1) as the time discount rate. The maximum likelihood estimation of the cardinal proxy values of the discount rate was applied, derived from the categorical responses in the questionnaire [18]. The proxy time discount rate (discrate) was standardised and averaged across the three questions. Respondents who switched their choices more than once, switched their choice in the category when given an interest rate of 5% or 0%, and/or who chose A for all categories (never switching to B) were considered to be irrational and their data were excluded from the analyses.

Hyperbolic time discounting was measured, comparing time discount rates for Q2 and Q3, following a methodology used in previous study [13]. By definition, if a respondent had a lower rate for Q3 (in a longer future) than for Q2 (in a near future), he or she could be classified as having a hyperbolic tendency. However, the decline in time discount rate in the future is typically not prominent beyond a six-month time frame [12]. Those who had a discount rate for Q3 lower than that for Q2 were classified as having a hyperbolic tendency in this study; otherwise participants were classified as having a non-hyperbolic tendency [13]. The hyperbolic tendency was set as missing if time discount rate either for Q2 or Q3 was missing. In a previous study, time discount score was significantly associated with debt holdings and obesity [13], indicating a piece of evidence for validity of the measure. The score of Q2 (i.e., an interest rate at which a respondent switch from the option A to B) was used as a control variable for the analyses on hyperbolic time discounting.

For a sensitivity analysis of the respondents who were excluded from the analyses for time discounting, five groups were prepared on the basis of irrational and missing responses to the scale and compared with the sample used for the main analysis on time preference; (a) an irrational group 1 (IR1) who selected reward in distant future that was lower (− 5% gain) than one in near future; (b) an irrational group 2 (IR2) who selected reward in distant future without no (0%) gain compared to one in near future; (c) an irrational group 3 (IR3) who kept selecting reward in near future despite of maximum gain to be obtained in distant future; (d) a switcher group (SW) who switched near and distant future rewards multiple times; (e) a missing group who had a missing response on any of three questions on time preference.

#### Personal values at present and at age 15

Personal values (value priorities and degrees of commitment to the values) at present were measured with the wave 3 questionnaire. To measure value priorities, we used a list of 11 values [6, 7, 34]: not bothering others, being evaluated by others, having and keeping a belief, succeeding economically, improving society, exploring what you are interested in, having influence on society, pursuing active challenges, cherishing family and friends, graduating from a famous school and maintaining a stable life (see Additional file 1: Appendix 2). Respondents were asked to report their value priorities at the time of the survey (‘How important do you think the following values are in your present life?’), rating each on a seven-point Likert scale (1 = Not at all important, 7 = Very important).

Commitment to value was measured via the Personal Values Questionnaire II (PVQ-II) [35]. The original version consists of nine items, but one item was dropped for the Japanese PVQ-II [36, 37]. Each item was rated on a five-point Likert scale to calculate the total score, with a higher score indicating greater commitment to a value. The internal consistency and concurrent and structural validity have been tested for the Japanese version [36, 37].

Personal values (both areas of priority vales and commitment to value) at age 15 were also measured based on a retrospective recall in the wave 3 survey. We set a time point for the recall of personal value at age of 15, when both time preference [38] and personal values are considered stable in adolescence [39]. Respondents were asked to recall their value priorities at age 15 (‘When you were 15–16 years old, how important did you think the following values were in your life?’) using the same list of 11 value areas, rating each on a seven-point Likert scale (1 = Not at all important, 7 = Very important). We modified the PVQ-II questions to the past tense and asked participants to rate their commitment to a value that was most important for them in their age of 15 [34]. Cronbach’s alpha coefficients for the PVQ-II were 0.711 and 0.741 for age 15 and adulthood, respectively.

Several unpublished studies of Japanese adults reported evidence for reliability and validity of the measures of areas of priority values. In a test-retest reliability study of areas of priority values at age 15, the scores reported twice with a two-week interval correlated moderately (ranging from 0.533 to 0.705 in intraclass correlations, ICCs), with one exception (0.372 in ICC for cherishing familiar people), while test-retest reliability statistic was not available for areas of priority values at present. For validity, the agreement (ICCs) between corresponding areas of priority values at age 15 and at present were greater for those who reported that their personal values were same since age 15 than for those who reported that these were different. The results indicate that areas of priority values at age 15 and at present can be measured separately by using the present questionnaire. On the other hand, for the PVQ-II, a pilot study of 500 adults in Japan reported acceptable levels of internal consistency and factor-based validity for the age-15 version and for the present version [34]. Cronbach’s alpha coefficients for the age-15 version and the present version of the PVQ-II were 0.680 and 0.732, respectively, in the present sample. The agreement (ICCs) between PVQ-II scores at age 15 and at present were greater for those who reported that their personal values were same since age 15 (0.596) than for those who reported that these were different (0.385), again indicating validity of asking these questions separately for age 15 and present.

#### Covariates

Sex, age, educational attainment, job status, and marital status were measured in the questionnaire as part of the wave 1 survey. We did not use household income as a covariate, because approximately 20% of respondents had missing values [18]. As this factor may be associated with personal values at age 15, two indicators of childhood socioeconomic status were measured in the questionnaire during the wave 1 survey. Parental education was defined as the highest educational attainment among respondents’ parents; this was dichotomised into university graduate or higher and less than university education. Economic hardship at age 15 was assessed by a question asking about the household economic situation when a respondent was 15 years old (with a 5-point scale from difficult to affluent), and dichotomised into yes (experiencing hardship) or no.

### Statistical analysis

Average scores of commitment to value (PVQ-II) and priority values as well as of time discount rate and hyperbolic time discount rate, were calculated. A linear regression analysis was conducted for the commitment to value score and a score for each priority value on time discount rate (crude), additionally adjusting for sex and age groups (sex- and age-adjusted), and further adjusting for educational attainment, job status, marital status, parental education, and economic hardship at age 15 (fully adjusted). A similar linear regression analysis was conducted of the commitment to value score and a score for each priority value on the dichotomous score on hyperbolic time discount rate (crude), additionally adjusting for the response to Q2 as a proxy of time discount rate, sex, and age groups (sex, age-, and base rate-adjusted), and further adjusting for educational attainment, job status, marital status, parental education, and economic hardship at age 15 (fully adjusted).

For the sensitivity analyses on the irrational and missing groups, scores of priority values and commitment to value were compared among the six groups (i.e., the sample for the main analysis, IR1–3, SW, and missing groups) for crude (one-way analysis of variance), sex- and age-adjusted and fully-adjusted models (analysis of variance).

The *p*-value for significance was set at < 0.05. However, considering multiple significant testing for the analysis for 11 priority values, we reset *p* for significance at < 0.0045 (=0.05/11), applying Bonferroni’s correction method. All statistical analyses were conducted by suing IBM SPSS version 26.

## Results

### Characteristics of the sample

After excluding respondents who had a missing response in demographic variables and indicators of personal value (*n* = 303), those with irrational responses to the time discount scale (IR1, *n* = 75; IR2, *n* = 143; SW, *n* = 51; and IR3, *n* = 176) and those with any missing response on Q1, Q2 or Q3 (*n* = 159), data from 1880 respondents were subject to further analysis on time discount (discrate). Similarly, after excluding the same respondents with missing in demographic variables and irrational responses to the time discount scale, and a missing response to Q1 (*n* = 81), data from 1958 were used in the analyses on hyperbolic time discount. About half of the sample included females; most participants were middle-aged or older, currently working and married; about half were university graduates (Table 1). Time discount rate ranged from − 0.390 to 4.234, with the highest frequency at the minimal value (24.7%). About one in 20 respondents had a tendency of hyperbolic time discounting. The average (SD) for the Q2 variable used as a control in the analyses of hyperbolic time discount was 7.42 (19.19). The score of commitment to value at present and at age 15 was almost normally distributed. Most scores for areas of priority values were left-skewed.

**Table 1:** Demographic characteristics, time preference indicators, and personal values at age 15 and at present in a community adult sample in Japan

	Sample used for analysis on discrate (*N* = 1880)				Sample used for analysis on hyperbolic (*N* = 1958)			
	n	%	Mean	SD	n	%	Mean	SD
Sex (male)	1048	55.7%			1099	58.5%		
Age								
20–39	385	20.5%			401	20.5%		
40–49	800	42.6%			824	42.1%		
50–60	695	37.0%			733	37.4%		
Educational attainment								
Junior high	50	2.7%			54	2.8%		
High school graduate	331	17.9%			351	17.9%		
Some college	642	34.8%			676	34.5%		
University graduate	857	46.4%			799	40.8%		
Job status (working)	1602	86.7%			1668	85.2%		
Marital status (married)	1433	77.6%			1493	76.3%		
Parental education (university or higher)	702	38.0%			733	37.4%		
Economic hardship at age 15	352	19.1%			365	18.6%		
Personal value:^a^								
Commitment to value at age 15 (16–40)			26.52	4.72			26.51	4.73
Area of priority value at age 15 (1–7)								
Not bothering others			5.62	1.33			5.62	1.33
Being evaluated by others			4.91	1.38			4.90	1.38
Having and keeping a belief			4.83	1.39			4.83	1.40
Economically succeeding			4.23	1.52			4.23	1.52
Improving society			3.77	1.43			3.76	1.44
Exploring what you were interested in			5.16	1.38			5.16	1.39
Having influence on society			3.27	1.40			3.27	1.41
Actively challenging			4.50	1.41			4.49	1.41
Cherishing familiar people			5.55	1.23			5.55	1.24
Graduating from a famous school			4.32	1.66			4.31	1.66
Maintaining a stable life			4.88	1.41			4.88	1.41
Commitment to value at present (16–40)			28.78	4.17			28.76	4.18
Area of priority value at present (1–7)								
Not bothering others			6.25	0.88			6.25	0.88
Being evaluated by others			4.77	1.31			4.77	1.31
Having and keeping a belief			5.56	1.12			5.56	1.12
Economically succeeding			5.35	1.09			5.36	1.08
Improving society			5.00	1.14			5.00	1.14
Exploring what you were interested in			5.41	1.13			5.40	1.14
Having influence on society			3.81	1.34			3.81	1.34
Actively challenging			5.24	1.21			5.24	1.22
Cherishing familiar people			6.47	0.84			6.47	0.84
Graduating from a famous school			4.14	1.56			4.13	1.57
Maintaining a stable life			6.11	0.92			6.11	0.92
Time preference								
Discrate			−0.07	0.67			NA^b^	NA^b^
Hyperbolic	72	3.8%			78	4.0%		

^a^ Score ranges in the parentheses

^b^ Could not calculated due to missing responses among 78 respondents

### Inter-correlations among variables

Inter-correlations among sociodemographic variables, personal values at present, and time preference are shown in the Additional file 2: Appendix Table 1. Women tended to have higher priorities for values at present on not bothering others, being evaluated by others, cherishing familiar people, graduating from a famous school, and maintaining a stable life, but less on economically succeeding, exploring what you were interested in, and having influence on society. Older respondents tended to have higher priorities for values at present on not bothering others, economically succeeding, improving society, having influence on society, graduating from a famous school, and maintaining a stable life, but lower scores of commitment to value. Women, older, and more educated, and married respondents reported low scores of time discount rate.

The scores of commitment to value at present and at age 15 correlated moderately (*r* = 0.471, *p* < 0.01) (Additional file 2: Appendix Table 2). The scores of a same priority value at age 15 and at present correlated moderately (rs, 0.353 on average, ranging from 0.257 to 0.480, all *p* < 0.01).

### Association between time preference and personal value at present

The score of commitment to value at present was not significantly associated with time discount score or hyperbolic time discounting, before or after adjusting for the covariates (Table 2). Time discount rate (discrate) was significantly and negatively associated with score for value priority of maintaining a stable life (*p* < 0.0045, Bonferroni’s correction). Time discount rate was significantly and negatively associated with value priority on economically succeeding and cherishing familiar people after adjusting for all covariates (*p* < 0.05), which did not reach at *p* < 0.0045 (Bonferroni’s correction). Time discounting or hyperbolic time discounting was not significantly associated with any other area of value priority at present.

**Table 2:** Association between time preference and personal values (commitment to value and area of priority values) at present (in adulthood) assessed in a community adult sample of Japan: Multiple linear regression analysis^a^

	Crude				Sex & age adjusted^b^				Fully adjusted^c^			
	b	SE	beta	p	b	SE	beta	p	b	SE	beta	p
Discrate (*N* = 1880)												
Commitment to value	−0.273	0.143	− 0.044	0.056	− 0.294	0.144	− 0.047	0.042	− 0.161	0.144	− 0.026	0.263
Not bothering others	−0.034	0.030	−0.026	0.264	−0.023	0.030	−0.018	0.444	−0.028	0.031	− 0.021	0.367
Being evaluated by others	−0.029	0.045	− 0.015	0.515	−0.060	0.045	− 0.031	0.187	− 0.049	0.046	−0.025	0.281
Having and keeping a belief	−0.011	0.038	−0.007	0.772	−0.013	0.039	−0.008	0.730	0.003	0.039	0.002	0.932
Economically succeeding	−0.085	0.037	−0.053	0.023	−0.100	0.038	−0.062	0.008	−0.089	0.038	−0.055	0.019
Improving society	−0.087	0.039	−0.052	0.025	−0.062	0.039	−0.037	0.113	−0.046	0.040	−0.027	0.250
Exploring what you were interested in	0.011	0.039	0.007	0.777	0.007	0.039	0.004	0.858	0.022	0.040	0.013	0.573
Having influence on society	− 0.013	0.046	−0.007	0.775	− 0.024	0.046	−0.012	0.609	−0.020	0.047	−0.010	0.669
Actively challenging	−0.072	0.041	−0.040	0.082	−0.081	0.042	−0.045	0.054	−0.064	0.042	−0.036	0.131
Cherishing familiar people	−0.112	0.029	−0.090	< 0.001*	−0.087	0.029	−0.070	0.002*	−0.075	0.029	−0.060	0.009
Graduating from a famous school	−0.149	0.053	−0.065	0.005	−0.120	0.054	−0.052	0.027	−0.065	0.054	−0.028	0.223
Maintaining a stable life	−0.147	0.031	−0.107	< 0.001*	−0.126	0.031	−0.092	< 0.001*	− 0.114	0.032	− 0.084	< 0.001*
Hyperbolic (*N*=1,958)												
Commitment to value	0.016	0.484	0.001	0.974	0.048	0.484	0.002	0.921	−0.130	0.479	−0.006	0.787
Not bothering others	−0.057	0.101	−0.013	0.572	−0.057	0.101	−0.013	0.577	−0.051	0.101	−0.012	0.612
Being evaluated by others	0.219	0.151	0.033	0.148	0.240	0.151	0.036	0.113	0.239	0.152	0.036	0.116
Having and keeping a belief	−0.148	0.129	−0.026	0.252	−0.149	0.129	−0.026	0.248	−0.185	0.130	−0.032	0.154
Economically succeeding	0.011	0.125	0.002	0.928	0.032	0.125	0.006	0.797	0.008	0.125	0.001	0.952
Improving society	−0.098	0.132	−0.017	0.456	−0.105	0.132	−0.018	0.426	−0.125	0.132	−0.021	0.345
Exploring what you were interested in	0.034	0.132	0.006	0.797	0.033	0.133	0.006	0.803	0.024	0.133	0.004	0.856
Having influence on society	−0.169	0.155	−0.025	0.278	−0.164	0.155	−0.024	0.291	−0.185	0.156	−0.027	0.236
Actively challenging	−0.004	0.141	−0.001	0.979	0.002	0.141	< 0.001	0.986	−0.015	0.141	−0.002	0.913
Cherishing familiar people	−0.149	0.097	−0.035	0.124	−0.153	0.096	−0.036	0.110	−0.171	0.095	−0.040	0.073
Graduating from a famous school	−0.101	0.181	−0.013	0.577	−0.097	0.181	−0.012	0.591	−0.154	0.179	−0.019	0.391
Maintaining a stable life	−0.058	0.106	−0.012	0.587	−0.061	0.105	−0.013	0.562	−0.069	0.106	−0.015	0.514

* Significant at *p* < 0.0045 (Bonferroni’s correction) for priority area of value

^a^ Regression of commitment to values or each area-specific score of priority values on time preference, and other covariates; standard regression coefficient (b), standard error (SE), and ps for significance are shown

^b^ Adjusted for sex and age groups in the analysis of time discount (discrate); adjusted for sex, age groups, and the response to Q2 in the analysis of hyperbolic time discount

^c^ Additionally adjusted for educational attainment, job status, marital status, parental education, and economic hardship at age 15

### Association between time preference and personal value at age 15

Hyperbolic time discounting was significantly and negatively associated with scores of commitment to value at age 15 after adjusting for the covariates (*p* = 0.046) (Table 3), while time discount rate was not. Time discount rate (discrate) was significantly and negatively associated with scores for value priority of cherishing familiar people, graduating from a famous school, and maintaining a stable life, after adjusting for all covariates (*p* < 0.05), and hyperbolic time discounting was associated with having influence on society(*p* = 0.045). But None of these associations did not reach at *p* < 0.0045 (Bonferroni’s correction). Time discount score or hyperbolic time discounting was not significantly associated with any other area of value priority at age 15.

**Table 3:** Association between time preference and personal values (commitment to value and area of priority values) at age 15 retrospectively assessed in a community adult sample of Japan: Multiple linear regression analysis^a^

	Crude				Sex & age adjusted^b^				Fully adjusted^c^			
	b	SE	beta	p	b	SE	beta	p	b	SE	beta	p
Discrate (*N* = 1880)												
Commitment to value	−0.229	0.162	−0.033	0.158	−0.294	0.164	−0.042	0.072	−0.183	0.165	−0.026	0.268
Not bothering others	−0.143	0.045	−0.073	0.002	−0.100	0.046	−0.051	0.029	−0.082	0.046	−0.041	0.076
Being evaluated by others	−0.137	0.047	−0.067	0.004	−0.120	0.047	−0.058	0.012	−0.081	0.047	−0.040	0.089
Having and keeping a belief	−0.017	0.048	−0.008	0.727	−0.019	0.048	−0.009	0.701	0.021	0.048	0.010	0.660
Economically succeeding	−0.047	0.052	−0.021	0.368	−0.065	0.052	−0.029	0.215	−0.059	0.053	−0.026	0.268
Improving society	−0.065	0.049	−0.030	0.188	−0.056	0.050	−0.026	0.259	−0.026	0.050	−0.012	0.607
Exploring what you were interested in	−0.028	0.047	−0.014	0.557	−0.068	0.048	−0.033	0.152	−0.042	0.048	−0.020	0.379
Having influence on society	−0.012	0.048	−0.006	0.806	−0.038	0.048	−0.018	0.435	−0.023	0.049	−0.011	0.643
Actively challenging	−0.023	0.048	−0.011	0.626	−0.031	0.049	−0.015	0.522	0.008	0.049	0.004	0.862
Cherishing familiar people	−0.138	0.042	−0.075	0.001	−0.109	0.042	−0.059	0.010	−0.100	0.043	−0.055	0.019
Graduating from a famous school	−0.275	0.057	−0.111	0.000	−0.251	0.057	−0.102	0.000	−0.136	0.053	−0.055	0.011
Maintaining a stable life	−0.192	0.048	−0.092	0.000	−0.172	0.049	−0.082	0.000	−0.137	0.049	−0.066	0.005
Hyperbolic (*N* = 1958)												
Commitment to value	−1.055	0.547	−0.044	0.054	−0.960	0.547	−0.040	0.079	−1.093	0.547	−0.045	0.046
Not bothering others	0.059	0.153	0.009	0.699	0.041	0.152	0.006	0.789	0.018	0.152	0.003	0.904
Being evaluated by others	0.019	0.160	0.003	0.905	0.017	0.159	0.002	0.915	−0.021	0.158	−0.003	0.896
Having and keeping a belief	−0.181	0.161	−0.025	0.261	−0.177	0.162	−0.025	0.273	−0.228	0.161	−0.032	0.155
Economically succeeding	−0.148	0.176	−0.019	0.398	−0.115	0.175	−0.015	0.512	−0.149	0.176	−0.019	0.397
Improving society	−0.099	0.166	−0.013	0.551	−0.088	0.166	−0.012	0.598	−0.130	0.165	−0.018	0.431
Exploring what you were interested in	−0.142	0.160	−0.020	0.375	−0.107	0.159	−0.015	0.503	−0.126	0.158	−0.018	0.426
Having influence on society	−0.318	0.162	−0.044	0.050	−0.294	0.162	−0.041	0.069	−0.326	0.162	−0.045	0.045
Actively challenging	−0.197	0.163	−0.027	0.226	−0.176	0.163	−0.024	0.280	−0.226	0.162	−0.031	0.163
Cherishing familiar people	0.069	0.143	0.011	0.628	0.062	0.142	0.010	0.662	0.048	0.142	0.008	0.734
Graduating from a famous school	0.204	0.191	0.024	0.286	0.233	0.191	0.028	0.221	0.113	0.176	0.013	0.522
Maintaining a stable life	0.196	0.163	0.027	0.230	0.201	0.163	0.028	0.217	0.148	0.162	0.021	0.361

* No variable was significant at *p* < 0.0045 (Bonferroni’s correction) for priority area of value

^a^ Regression of commitment to values or each area-specific score of priority values on time preference, and other covariates; standard regression coefficient (b), standard error (SE), and ps for significance are shown

^b^ Adjusted for sex and age groups in the analysis of time discount (discrate); adjusted for sex, age groups, and the response to Q2 in the analysis of hyperbolic time discount

^c^ Additionally adjusted for educational attainment, job status, marital status, parental education, and economic hardship at age 15

### Comparison of personal values among groups classified based on irrational, switching, and missing responses

Commitment to value at present was significantly different among the six groups classified on irrational, switching, and missing responses (*p* = 0.002) after adjusting all the covariates, with significantly lower average scores in the IR1 and missing groups (*p* < 0.001) (Additional file 2: Appendix Table 3). Among value priorities, improving society, having influence on society, and maintaining a stable life were significantly different among the groups (*p* < 0.0045, Bonferroni’s correction). For the post-hoc test of the difference from the sample used in the main analysis, the IR3 group showed significantly lower mean scores of graduating from a famous school (*p* < 0.0045, Bonferroni’s correction).

Commitment to value at age 15 was marginally significantly different among the six groups (*p* = 0.051) after adjusting all the covariates, with significantly lower average scores in the IR1 group (*p* = 0.004) (Additional file 2: Appendix Table 4). Among value priorities, not bothering others, having interest in society, cherishing familiar people, and graduating from a famous school were significantly different among the groups (*p* < 0.05), while the level of significance did not reach at *p* < 0.0045 (Bonferroni’s correction). For the post-hoc test of the difference from the sample used in the main analysis, the IR3 group showed significantly lower mean scores of not bothering others, cherishing familiar people, and graduating from a famous school (*p* < 0.0045, Bonferroni’s correction).

## Discussion

In general, the associations between personal value parameters and time preference indicators were scarce and small if any. However, the present study found that having a priority for personal value on maintaining a stable life at present and at age 15 was significantly and negatively associated with time discount rate in a sample of adult community residents in Japan. Having high commitment to value at age 15 was significantly and negatively associated with hyperbolic time discounting. This is the first evidence that time preference and personal values are associated. The effect sizes for these associations were relatively small (< 0.10 in a standardized coefficient) and there was no other significant association observed. The present results suggest that time discounting plays a limited role in choosing priority values. However, it is interesting to note that value priorities and commitment to value were associated with two distinct time discounting indicators. The present study could be a starting point for more rigorous future investigations such as longitudinal cross-lag designs and/or more reliable measures of time preference.

Time discount rate was significantly and negatively associated with one value area, i.e. maintaining a stable life, at present. The similar pattern was observed for this value areas at age 15, while it was only marginally significant after applying Bonferroni’s correction. This value can be classified as one related to conservation-security according to the Schwartz’s classification of basic values [2]. People with a greater time discount rate tend to perceive a lower present value of future reward, and thus take risky behaviours [12]. Thus people with greater time discount are less likely to choose maintaining a stable life as their priority value. The same explanation may apply to the pattern that time discount rate was negatively associated with values areas of economically succeeding, cherishing familiar people, and graduating from a famous school, while these were non-significant after Bonferroni’s correction, and that respondents who kept selecting reward in near future despite of a maximum gain to be obtained in distant future (the irrational group 3 who was supposed to have a high time discount rate) showed lower scores of cherishing familiar people, graduating from a famous school, and maintaining a stable life in general. The other interpretation is, as time discounting decreases along with age as maturation of prefrontal regulatory regions [22, 25], choosing a priority for the value of maintaining a stable life may have an effect on decreasing time discounting during the development from adolescence to adulthood, possibly via the selection of a stable and secure life. Future research is needed to determine if the time discounting tendency affects the development of personal value priority on security or a value on security affect the development of a less (or more matured) tendency of time discounting. Respondents who selected reward in distant future with no gain (the IR2) and who kept selecting reward in near future despite of a maximum gain to be obtained in distant future (the IR3) tended to have lower scores of commitment to value at age 15 and at present, respectively, compared to the final sample in the analysis. The pattern was inconsistent with the findings based on the regression analyses of the main sample. It would be interesting to investigate further if these groups are extreme cases on a one-dimensional scale of time discounting or they have unique characteristics that could affect the development of personal values.

Hyperbolic time discounting was significantly and negatively associated with commitment to value at age 15. Hyperbolic time discounting is a pattern that time discount rates are not constant over time. Thus, individuals with this tendency tend to make choices that are inconsistent over time [12]. This tendency may prevent people from developing a strong commitment to a particular value, since the development of commitment to a specific value requires a long-term, consistent psychological process aligned to the selected value. A similar explanation could apply to the finding that hyperbolic time discounting was negatively and non-significantly (after Bonferroni’s correction) associated with having influence on society. On the other hand, hyperbolic time discounting was not significantly associated with commitment to value at present. The association between these two constructs may be more prominent when the commitment to value first formed in adolescence. The association may become weaker when the commitment to value is affected or compromised through life experiences in a later life. Most plausible hypotheses raised by the present findings are that (1) hyperbolic time discounting tendency could affect only the early development of commitment to value in adolescence or (2) an early establishment of commitment to a value could help people develop a less tendency of hyperbolic time discounting. i.e. a more rational tendency in intertemporal choices, in a later life. Future research may be promising if it tests these hypotheses in a longitudinal study of adolescents or young adults, because these transition is likely to occur in these stages of life [22, 30].

In sum, time discounting was significantly associated with one area of priority values (a stable life), while it was not with commitment to value. On the other hand, hyperbolic time discounting was significantly associated with commitment to value, while it was not with any area of priority values. Although further research is needed to conform if this pattern exists, selecting areas of priority values and having high commitment to a value regardless of what the value is may be associated with different types of tendencies in intertemporal choices, i.e., time discounting and hyperbolic time discounting, respectively. Previous studies indicate that time discounting and hyperbolic discounting may stem from a combined activity between different neural networks in brain [21, 24, 26]. The findings might contribute to developing a further hypothesis on behavioural and neuroeconomic determinants or influences of personal value. For instance, there may be specific sets of a dimension of personal value (area of priority value or commitment to value), time preference (time discounting or hyperbolic time discounting), and type of brain function (impulsive or rational brain). The connections among these variables need to be studied in a perspective that personal values and time preference may dynamically change over time, in particular, between adolescence and young adult, as suggested by previous neuroimaging studies [22, 25] and also from moderate correlations between personal values at age 15 and in adulthood observed in this study. However, the effect sizes (standardized correlation coefficients) for the association between personal value indicators and time preference were relatively small (< 0.10) even where these associations were statistically significant. The associations between personal values and time preference may be small if any.

### Limitations

Other limitations should be considered in interpreting the findings of this study. First, the initial response rate was low; only half of the initial respondents completed the wave 3 questionnaire, and respondents who disclosed irrational options on time discount questions were excluded. These may have induced selection bias if people with both high time preference and low personal values were less likely to participate in the study. Second, some information bias may exist. For instance, time preference may be associated with inaccurate recall of personal values at age 15. This could result in both over- and under-estimation of the association between these factors. Third, a common factor that was not measured in this study, such as intelligence, may confound the association. Fourth, reliability and validity of measures of time preference were still not very clear. Test-retest reliability for the areas of priority values and commitment of value at age 15 was moderate; validity of these measures are still not fully addressed. The scale used to measure time preference is not fully validated yet, while there is plenty of evidence showing its construct validity [13, 15, 18]. Measurement error may occur in the retrospective assessment of personal values, as well as in the assessment of time preference. Furthermore, our classification of the hyperbolic time discount group yielded a much smaller proportion of hyperbolic time discount compared to previous studies using choice-based measures. e.g., 62.1% in a study in Japan by Ikeda et al., 2010 [13]. This is attributable to the different time frame used in the scales. Ikeda et al. [13] used two questions for immediate future choice (i.e., 2 days or 9 days) and a more distant future choice (i.e., 90 days or 97 days); we used much longer time frames for recent future choice (i.e., 1 month or 13 months) and a more distant future choice (i.e., 13 months or 25 months). It was observed that the time discount rate became smaller when the time intervals are longer [40]. The method used in this study may not be fully sensitive to identify the hyperbolic time discount. However, the present study indicated the association between time preference and personal value in a large-scale population-based study. This warrants further research on the association between these two value-related constructs.

## Conclusions

There may be an association between time preference and personal values, although the direction of the association needs to be studied in future research. Time discounting and hyperbolic time discounting may be associated with different aspects of personal values, i.e., area of priority values and commitment to value, respectively.

## Supplementary information

**Additional file 1.**

**Additional file 2.**

## Data Availability

Data are available from the Data Management Committee of the Japanese Study on Stratification, Health, Income, and Neighborhood (J-SHINE) for researchers who meet the criteria for access to data.

## Abbreviations

J-SHINE: The Japanese Study on Stratification, Health, Income and Neighbourhood
IR1: The irrational group 1
IR2: The irrational group 2
IR3: The irrational group 3
SW: The switcher group
PVQ-II: Personal Values Questionnaire II
ICC: Intraclass correlation
