# Supplementary material for: Time preference and personal value: a population-based cross-sectional study in Japan
Source: BMC Psychol. 2020 Aug 17;8:85. doi: 10.1186/s40359-020-00458-6 (PMC7433046; doi:10.1186/s40359-020-00458-6)
Supplement: Supplementary file 1 — Additional file 1. [file 40359_2020_458_MOESM1_ESM.docx]

Appendix 1. The scale of time preference (time discounting and hyperbolic time discounting)

Adopted from the wave 1 questionnaire (2010) of the Japanese Study on Stratification, Health, Income and Neighbourhood (J-SHINE) (Takagi et al., 2016),

Q1

These next questions will ask for your views on money and asset management.

Suppose that in 1 month from now you can get 10,000 yen. You could choose to take this money, but if you choose to not take the money and wait for another year, that is 13 months from now, you would get a different amount of money.

Let the option of taking the 10,000 yen in one month from now be (A), and the amount that you could get if you wait 13 months be (B).

Looking at the following amounts, which would you choose in each case?

| 1 month from now (A) | Which would you choose? | | 13 months from now (B) | (B) interest rate (annual rate) |
| --- | --- | --- | --- | --- |
| 10,000 | A | B | 9,500 | -5% |
| 10,000 | A | B | 10,000 | 0% |
| 10,000 | A | B | 10,200 | 2% |
| 10,000 | A | B | 10,400 | 4% |
| 10,000 | A | B | 10,600 | 6% |
| 10,000 | A | B | 11,000 | 10% |
| 10,000 | A | B | 12,000 | 20% |
| 10,000 | A | B | 14,000 | 40% |
| 10,000 | A | B | more than 14,000 | > 40% |

Q2

Now then, suppose that in 1 month from now you can get 1 million yen. You could choose to take this money, but if you choose to not take the money and wait for another year, that is 13 months from now, you would get a different amount of money.

Let the option of taking the 1 million yen in one month from now be (A), and the amount that you could get if you wait 13 months be (B).

Looking at the following amounts, which would you choose in each case?

| 1 month from now (A) | Which would you choose? | | 13 months from now (B) | (B) interest rate (annual rate) |
| --- | --- | --- | --- | --- |
| 1,000,000 | A | B | 950,000 | -5% |
| 1,000,000 | A | B | 1,000,000 | 0% |
| 1,000,000 | A | B | 1,001,000 | 0. 1% |
| 1,000,000 | A | B | 1,005,000 | 0. 5% |
| 1,000,000 | A | B | 1,010, 000 | 1% |
| 1,000,000 | A | B | 1,020,000 | 2% |
| 1,000,000 | A | B | 1,060,000 | 6% |
| 1,000,000 | A | B | 1,100,000 | 10% |
| 1,000,000 | A | B | more than 1,100,000 | > 10% |

Q3

Now then, suppose that in 13 months from now you can get 1 million yen. You could choose to take this money, but if you choose to not take the money and wait for another year, that is 25 months from now, you would get a different amount of money.

Let the option of taking the 1 million yen in 13 months from now be (A), and the amount that you could get if you wait 25 months be (B).

Looking at the following amounts, which would you choose in each case?

| 13 months from now (A) | Which would you choose? | | 25 months from now (B) | (B) interest rate (annual rate) |
| --- | --- | --- | --- | --- |
| 1,000,000 | A | B | 950,000 | -5% |
| 1,000,000 | A | B | 1,000,000 | 0% |
| 1,000,000 | A | B | 1,001,000 | 0. 1% |
| 1,000,000 | A | B | 1,005,000 | 0. 5% |
| 1,000,000 | A | B | 1,010, 000 | 1% |
| 1,000,000 | A | B | 1,020,000 | 2% |
| 1,000,000 | A | B | 1,060,000 | 6% |
| 1,000,000 | A | B | 1,100,000 | 10% |
| 1,000,000 | A | B | more than 1,100,000 | > 10% |

Appendix 2. The scales of personal values (value priorities) at present and at age 15

Adopted from the wave 3 questionnaire (2017) of the Japanese Study on Stratification, Health, Income and Neighbourhood (J-SHINE); originally developed by Watanabe, Kawakami, & Nishi (2020)

**About your values (15-16 years old)**

We will ask about your thoughts when you were 15-16 years old. Please answer the following questions remembering the time.

| When you were *15-16 years old*, how important did you think the following *values* in your life? | | Not at all | Not very important | A little not important | Neutral | A little important | Somewhat important | Very important |
| --- | --- | --- | --- | --- | --- | --- | --- | --- |
| 1 | Not bothering others | 1 | 2 | 3 | 4 | 5 | 6 | 7 |
| 2 | Being evaluated by others | 1 | 2 | 3 | 4 | 5 | 6 | 7 |
| 3 | Having and keeping a belief | 1 | 2 | 3 | 4 | 5 | 6 | 7 |
| 4 | Economically succeeding | 1 | 2 | 3 | 4 | 5 | 6 | 7 |
| 5 | Improving society | 1 | 2 | 3 | 4 | 5 | 6 | 7 |
| 6 | Exploring what you were interested in | 1 | 2 | 3 | 4 | 5 | 6 | 7 |
| 7 | Having influence on society | 1 | 2 | 3 | 4 | 5 | 6 | 7 |
| 8 | Actively challenging | 1 | 2 | 3 | 4 | 5 | 6 | 7 |
| 9 | Cherishing familiar people | 1 | 2 | 3 | 4 | 5 | 6 | 7 |
| 10 | Graduating a famous school | 1 | 2 | 3 | 4 | 5 | 6 | 7 |
| 11 | Maintaining a stable life | 1 | 2 | 3 | 4 | 5 | 6 | 7 |

**About your values (current)**

We will ask about your current thoughts. Please answer the following questions.

| *Now* how important do you think the following values in your life? | | Not at all | Not very important | A little not important | Neutral | A little important | Somewhat important | Very important |
| --- | --- | --- | --- | --- | --- | --- | --- | --- |
| 1 | Not bothering others | 1 | 2 | 3 | 4 | 5 | 6 | 7 |
| 2 | Being evaluated by others | 1 | 2 | 3 | 4 | 5 | 6 | 7 |
| 3 | Having and keeping a belief | 1 | 2 | 3 | 4 | 5 | 6 | 7 |
| 4 | Economically succeeding | 1 | 2 | 3 | 4 | 5 | 6 | 7 |
| 5 | Improving society | 1 | 2 | 3 | 4 | 5 | 6 | 7 |
| 6 | Exploring what you are interested in | 1 | 2 | 3 | 4 | 5 | 6 | 7 |
| 7 | Having influence on society | 1 | 2 | 3 | 4 | 5 | 6 | 7 |
| 8 | Actively challenging | 1 | 2 | 3 | 4 | 5 | 6 | 7 |
| 9 | Cherishing familiar people | 1 | 2 | 3 | 4 | 5 | 6 | 7 |
| 10 | Graduating a famous school | 1 | 2 | 3 | 4 | 5 | 6 | 7 |
| 11 | Maintaining a stable life | 1 | 2 | 3 | 4 | 5 | 6 | 7 |
